# Clinical warning signs of life-threatening hematochezia in neurosurgical patients with long-term bed rest: Three cases report

**DOI:** 10.1097/MD.0000000000022471

**Published:** 2020-09-25

**Authors:** Ji-Ho Jung, Yong-Hwan Cho, Man-Seok Park, Sung-Pil Joo

**Affiliations:** a Department of Neurosurgery; b Department of Neurology, Chonnam National University Hospital and Medical School, Gwangju, Republic of Korea.

**Keywords:** hematochezia, intensive care unit, neurosurgery, stress

## Abstract

**Rationale:**

Patients with long term bed rest in intensive care unit after neurosurgery could experience splanchnic hypoperfusion. These patients have several other medical conditions that exacerbate splanchnic hypoperfusion during treatment and the splanchnic hypoperfusion could result in “stress-induced intestinal necrosis”, which could cause massive hematochezia. We report here the experience of life-threatening hematochezia in 3 patients who underwent brain surgery in our institution.

**Patients concerns:**

One female patient (72-year-old) and 2 male patients (58- and 35-year-old) were admitted to our institution because of traumatic intracerebral hemorrhage, subarachnoid hemorrhage due to a ruptured anterior communicating artery, and subarachnoid hemorrhage with unknown cause respectively. All patients underwent emergency brain surgery for diagnosis and treatment. After surgery, they all experienced long-term bed rest in intensive care unit. Hematochezia occurred on postoperative day 15, 17, and 49, respectively.

**Diagnoses:**

All of the patients were assessed by abdomen/pelvis computed tomography and underwent a colonoscopy.

**Interventions:**

The female patient underwent embolization through pelvic arteriography and epinephrine injection through colonoscopy, but a total colectomy and ileostomy was performed due to refractory hematochezia. 58-year-old male patient had a laparoscopic ileostomy for the bowel rest. The other patient underwent nil per os and conservative treatment for 2 weeks.

**Outcomes:**

The female patient was discharged without further treatment plan, 58-year-old male patient survived after laparoscopic ileostomy, while the other patient survived after 2 weeks of nil per os.

**Lesson:**

Abdominal symptoms, such as hematochezia, should be actively managed in neurosurgical patients who are undergoing long-term bed rest in an intensive care unit under physiologically stressful medical conditions.

## 1 Introduction

Patients who undergo brain surgery often need prolonged intensive care unit (ICU) management. The patients treated in ICU may suffer from a number of different symptoms during their treatment. In particular, gastrointestinal (GI) tract dysfunction is quite common. Many GI tract complications, such as diarrhea, constipation, stool impaction, infection, and hematochezia, can occur during long-term hospitalization. According to Reintam A et al, 59% of patients experience at least 1 GI symptom during their ICU stay.^[1]^ However, neurosurgeons tend to overlook GI tract symptoms, since their focus is on patients’ neurological changes and ICU patients are frequently unable to adequately communicate their symptoms. Additionally, there are currently no validated markers for assessing gut function,^[1,2]^ with some authors describing GI bleeding as the only credible symptom of GI track failure.^[1,3]^ Critical illness that needs brain surgery can cause splanchnic hypoperfusion, resulting in mesenteric ischemic necrosis, which in turn can manifest as hematochezia.^[4]^ We report here the experience of life-threatening hematochezia in 3 patients who underwent brain surgery in our institution between January 1, 2016 and December 31, 2017. By analyzing these events, we aim to evaluate the importance of abdominal symptoms, such as hematochezia, in the management of ICU patients in the neurosurgery department.

## 2 Case presentation

We reviewed the medical records of 1965 patients who underwent brain surgery between January 1, 2016 and December 30, 2017. Three patients who experienced massive hematochezia and were diagnosed with chronic ulcer by colonoscopy and biopsy findings were identified and selected for further analysis. One of the patients was discharged without further management plan, while 2 patients survived and recovered (Table 1). For these 3 cases, we made the diagnosis of “stress-induced bowel ischemia manifesting as hematochezia” after detailed consideration.

**Table 1:**
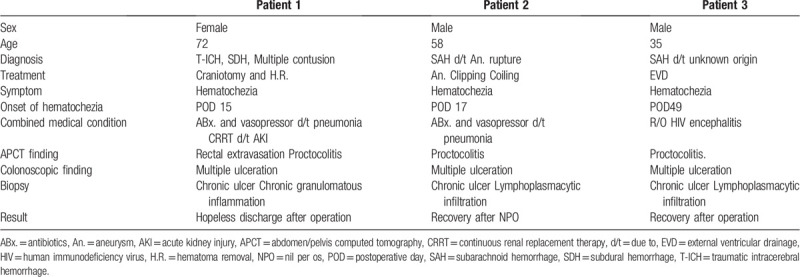
Summary of the reported cases.

### 2.1 Illustrative cases

**Figure 1:**
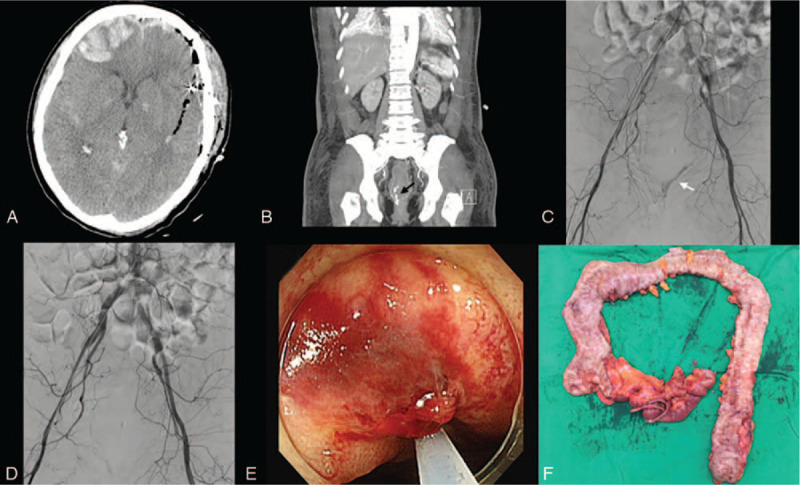
Case 1. A: Patient underwent craniotomy and removal of a hematoma due to traumatic intracerebral hemorrhage in the left temporal lobe. B: APCT revealed media extravasation at rectum (black arrow). C. Pre-angioembolization angiography shows extravasation of right middle rectal artery (white arrow). D: Extravasation of contrast media stopped following embolization with histoacryl and lipiodol. E: Following a recurrence of bleeding, colonoscopy reveled multiple ulcerative lesions with suspected exposed vessel. F: Total colectomy and end-ileostomy was performed for management of refractory hematochezia. APCT = abdomen/pelvis computed tomography.

A 72-year-old woman who had no past medical history was admitted to the emergency center in mental stupor after a fall. At the emergency room, Glasgow coma scale score of 10 was determined. Initial computed tomography (CT) revealed traumatic intracerebral haemorrhage in the left temporal lobe, frontal subdural hemorrhage, traumatic subarachnoid hemorrhage (SAH), and multiple hemorrhagic contusions. Emergency craniotomy and removal of the hematoma were performed. Following the procedure, the patient was admitted to the ICU, where she was treated with antibiotics for concomitant pneumonia and continuous renal replacement therapy (CRRT) for acute renal injury. She was on a mechanical ventilator and a vasopressor was administered due to the general condition, followed by sedative therapy for brain swelling. On the 15th day of the ICU treatment, massive hematochezia occurred. Abdomen/pelvis CT (APCT) revealed contrast media extravasation in the rectum, as well as proctocolitis and enteritis involving the pelvic small bowel loop. To treat the extravasation in the rectum, pelvic arteriography and embolization were performed. Bleeding recurred at postoperative day (POD) 27, requiring colonoscopy and administration of epinephrine. Despite these procedures, refractory hematochezia continued. The patient underwent total colectomy and ileostomy, after which she experienced sepsis due to peritonitis. The patient was discharged with no further treatment plan (Fig. 1).

**Figure 2:**
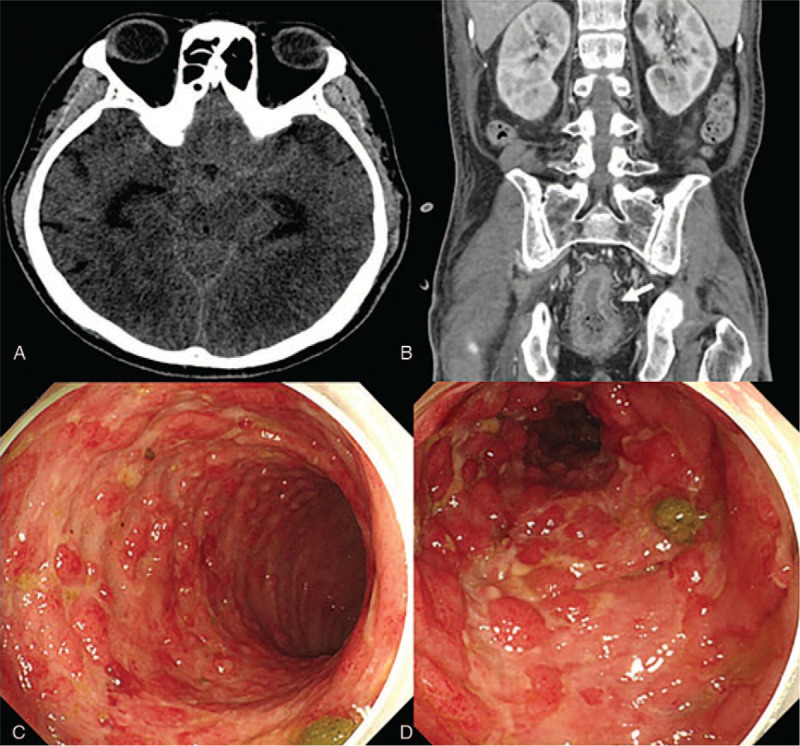
Case 2. A: SAH with no aneurysm sac. B: APCT revealed edematous wall thickening in distal sigmoid colon and rectum, suggesting proctocolitis (white arrow). C and D: Colonoscopy showed diffuse ulcerative lesions with mucosal friability. APCT = abdomen/pelvis computed tomography, SAH = subarachnoid haemorrhage.

A 35-year-old man positive for human immunodeficiency virus infection was admitted to our hospital with sudden paraparesis (grade III) with rigidity. A brain CT revealed SAH of unknown origin and spinal magnetic resonance imaging demonstrated intradural extramedullary mass on thoracic 11 to 12 level. The patient underwent cerebral angiography, which detected no aneurysm. There were no abnormal signs of somatosensory evoked potential, and results of nerve conduction study and electromyography were normal. Extra-ventricular drainage was performed to identify human immunodeficiency virus encephalitis, but ribonucleic acid polymerase chain reaction was negative in the analysis of the cerebrospinal fluid. At POD 49, hematochezia occurred. APCT showed proctocolitis, while colonoscopy revealed ulcerative lesions. The patient recovered after laparoscopic ileostomy for bowel rest. Chronic ulcer was confirmed by pathologic exam (Fig. 2).

**Figure 3:**
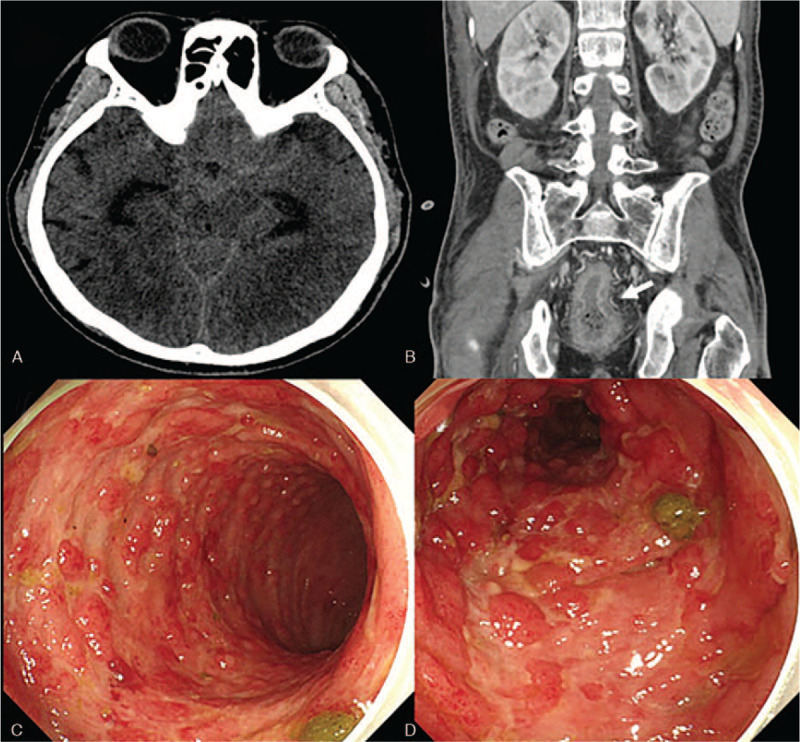
Case 3. A: APCT showed rectum and sigmoid colon wall thickening and enhancement suggestive of proctocolitis involving rectum and sigmoid colon (white arrows). B, C, and D: serial colonoscopy images. Multiple ulcerative lesions improved following NPO. APCT = abdomen/pelvis computed tomography, NPO = nil per os.

A 58-year-old man with ruptured anterior choroidal artery aneurysm underwent clipping. Ten days later, coil embolization was performed because of aneurysm recurrence, followed by administration of dual antiplatelet agents. In ICU, the patient was treated for sepsis with antibiotics and a vasopressor. Hematochezia was observed at POD 17. Colonoscopy showed multiple erosion. After 2 weeks of nil per os management and discontinuation of the antiplatelet agents, the patient recovered (Fig. 3).

## 3 Discussion

Delayed massive hematochezia occurred in 3 patients undergoing long-term ICU care following brain surgery. Two of the 3 patients underwent surgical treatment and 1 underwent 2 weeks of intense conservative treatment. One of the patients who underwent surgical treatment was discharged with no treatment plan, while the remaining surgically treated patient and the conservatively treated patient recovered fully. All of the studied patients exhibited the following common characteristics:

(1) experience of brain surgery;
(2) long-term bed rest in ICU;
(3) concomitant medical issue, such as infection or vasopressor usage;
(4) severe constipation and abdominal distension without early abdominal tenderness;
(5) proctocolitis finding in APCT;
(6) multiple ulcerative lesions on colonoscopy; and
(7) pathological examination suggesting the presence of a chronic ulcer.

The mucosal and submucosal layers of GI tract is vulnerable to ischemic changes because seventy percent of the mesenteric blood flow perfuses these layers.^[2]^ The extent of intestinal damage due to ischemia varies, ranging from mucosal lesions due to reversible ischemia to necrosis and perforation with transmural injury.^[5]^ Stress conditions, such as ICU care following brain surgery, can result in splanchnic hypoperfusion, which was shown to be linked with mesenteric ischemic necrosis.^[4]^ If intestinal necrosis or ulceration exposes the bowel lumen vessels and there is damage present on the exposed vessels, it can lead to massive hematochezia. Stress situations induce sympathetic nervous system excitation and vagal inactivation. Sympathetic excitation causes intestinal vasoconstriction and results in mucosal hypoxia, while vagal inactivation inhibits anti-inflammatory pathways, leading to intestinal injury.^[4,6–8]^ Approximately 3% to 4% of stress-induced mucosal damage was reported to result in clinically significant GI bleeding.^[9]^ The 3 patients in our study underwent long periods of ICU care following brain surgery, as well as concomitant medical conditions. Their bodies were subjected to a constant stress led to intestinal necrosis.

**Figure 4:**
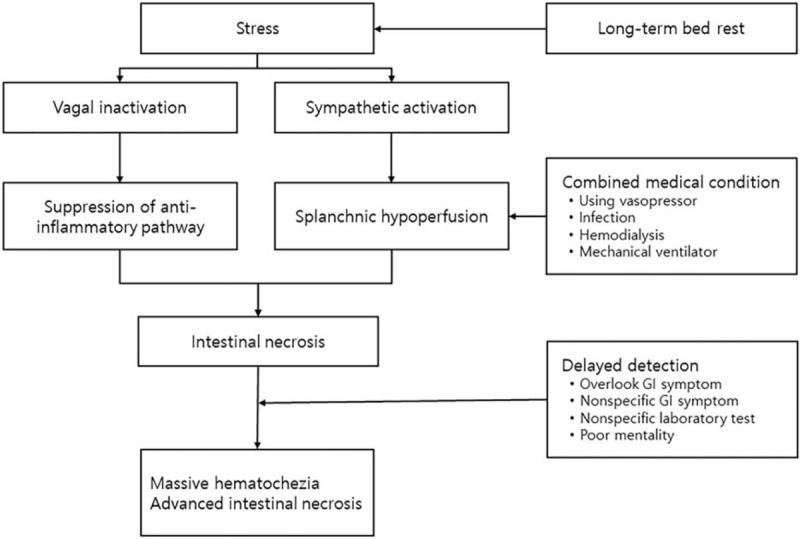
Overview of the development of stress-induced intestinal necrosis and hematochezia following neurosurgery in critically ill patients in ICU care with multiple concomitant medical conditions. ICU = intensive care unit.

A number of other concomitant medical conditions experienced by neurosurgical ICU patients, such as the use of vasopressors, sepsis, hemodialysis, and utilization of mechanical ventilators, can exacerbate splanchnic hypoperfusion. Vasopressors are commonly used to maintain cerebral perfusion during delayed vasospasm in subarachnoid hemorrhage caused by aneurismal rupture, septic shock, and neurogenic shock.^[10,11]^ Because vasopressors centralize the blood volume and increase blood supply to vital organs such as the brain and heart, they cause splanchnic hypoperfusion, which can exacerbate mesenteric ischemia.^[9,12]^ In patients with multiple trauma in neurosurgical ICU, acute kidney injury due to rhabdomyolysis, acute kidney injury due to use of mannitol to control intracranial pressure, refractory metabolic acidosis, and acute kidney injury in septic conditions may occur. In these situations, renal replacement such as conventional hemodialysis and CRRT cause hypovolemia and myocardial depression, leading to a decrease in splanchnic perfusion.^[13,14]^ Use of a mechanical ventilator was shown to cause splanchnic hypoperfusion (resulting from positive end-expiratory pressure [PEEP]), decrease cardiac output, activate the sympathetic system, and cause a systemic inflammatory response that results in GI tract damage (Fig. 4).^[9]^

Intestinal necrosis, which occurs in neurosurgical patients, may be detected late for the following reasons:

(1) neurosurgeons tend not to pay much attention to the patient's GI symptoms,
(2) chronic intestinal necrosis manifests as non-specific GI symptoms in the early phase,
(3) there are no specific laboratory tests for detecting mesenteric ischemia, and
(4) mentally impaired patients do not provide adequate descriptions of GI symptoms (Fig. 4).

In advanced intestinal necrosis, at the point at which neurosurgeons become aware of the severity of the GI symptoms, the patient can experience massive hematochezia and intestinal necrosis may have already progressed too far to treat easily. Gong et al reported 3 cases of stress-induced intestinal necrosis.^[15]^ Stress-induced intestinal necrosis is a process that progresses slowly and does not accompany abdominal pain in early stages. Accordingly, the 3 patients in this study exhibited signs of hematochezia on POD 15, 17, or 49, but did not complain of acute abdominal pain. Routine laboratory tests such as the measurements of lactate, lactate dehydrogenase, amylase, D-dimer, pH, and base excess have low specificity for diagnosing mesenteric ischemia.^[16]^ Recently, meta-analysis evaluating the use of serum intestinal fatty acid-binding protein for the diagnosis of acute intestinal ischemia reported a sensitivity of 0.80 and specificity of 0.85.^[17]^ However, the use of serum intestinal fatty acid-binding protein as a biomarker is still under study and clinical use is not common.

GI tract bleeding is associated with significantly higher mortality^[3]^ and the prognosis in patients with mesenteric ischemia is poor, with a lethal outcome found in up to 60% of patients.^[18,19]^ GI complications requiring surgical treatment, such as GI bleeding and perforation, were reported in 6.8% of neurosurgical patients, with 2.1%, patients dying of GI complications.^[20]^ In our study, 1 of the patients was discharged with no further treatment planned, 1 survived after laparoscopic ileostomy, and 1 survived after 2 weeks of Nil per os. Based on our observations, a number of recommendations can be made. First, if a patient is affected by concomitant medical conditions, he/she should be considered to be at risk of stress-induced intestinal necrosis. All 3 cases in our report presented with concomitant medical conditions, notably infection. Two of the 3 cases needed vasopressors. The patient who was discharged with no curative plan underwent treatment with CRRT, mechanical ventilator, vasopressor, and antibiotics due to septic shock. Second, non-essential use of vasoconstrictors should be prohibited. Third, normal intestinal environment should be maintained by adjusting the patient's bowel habits. Patients undergoing long-term bed rest tend to experience abdominal distension due to GI tract hypomotility. Worsening GI distension leads to a disruption in the blood supply, resulting in injury to the GI wall and thereby causing bacterial translocation, leading to peritoneal infection and septic conditions.^[4,9]^ Stress-induced intestinal necrosis is a relatively slow process, so it is difficult to predict the occurrence of GI failure in advance. Therefore, neurosurgeons should regularly check the GI symptoms of patients who are affected by physiological stress conditions.

## 4 Conclusion

Regeneration of the GI tract is inhibited in the conditions of physiological stress, and worsening of the situation can lead to severe colitis and ulceration. Patients with severe cerebral lesions undergo prolonged state of absolute bed rest in ICU care, tend to experience septic conditions with prolonged use of antibiotics and vasopressor agents, and are often kept on mechanical ventilation. In these situations, the patients with cerebral lesions are exposed to prolonged physiological stress. There are multiple factors that can delay the diagnosis of GI problems, including the focus of the treating physician on neurological changes, the inability of ICU patients to report symptoms, and the lack of validated markers for gut function. It is essential for physicians to pay close attention to the GI symptoms in critically ill patients following neurosurgery, particularly in patients with multiple concomitant medical conditions, and to actively cope with GI issues.

## Author contributions

**Conceptualization:** Sung-Pil Joo

**Data curation:** Ji-Ho Jung, Yong-Hwan Cho

**Supervisions:** Man-Seok Park, Sung-Pil Joo

**Writing:** Ji-Ho Jung
